# Fibulin-2: A Novel Biomarker for Differentiating Grade II from Grade I Meningiomas

**DOI:** 10.3390/ijms22020560

**Published:** 2021-01-08

**Authors:** Agbolahan A. Sofela, David A. Hilton, Sylwia Ammoun, Daniele Baiz, Claire L. Adams, Emanuela Ercolano, Michael D. Jenkinson, Kathreena M. Kurian, Mario Teo, Peter C. Whitfield, Felix Sahm, C. Oliver Hanemann

**Affiliations:** 1 Faculty of Health, Medicine, Dentistry and Human Sciences, The Institute of Translational and Stratified Medicine, University of Plymouth, The John Bull Building, Plymouth Science Park, Research Way, Plymouth PL6 8BU, UK; agbolahan.sofela@plymouth.ac.uk (A.A.S.); sylwia.ammoun@plymouth.ac.uk (S.A.); daniele.baiz@plymouth.ac.uk (D.B.); claire.adams@plymouth.ac.uk (C.L.A.); emanuela.ercolano@plymouth.ac.uk (E.E.); 2 South West Neurosurgery Centre, University Hospitals Plymouth NHS Trust, Derriford Road, Plymouth PL6 8DH, UK; peter.whitfield@nhs.net; 3 Cellular and Anatomical Pathology, University Hospitals Plymouth NHS Trust, Derriford Road, Plymouth PL6 8DH, UK; davidhilton@nhs.net; 4 Department of Neurosurgery, The Walton Centre NHS Foundation Trust, Lower Lane, Liverpool L9 7LJ, UK; michael.jenkinson@liv.ac.uk; 5 Institute of Translational Medicine and School of Medicine, University of Liverpool, Liverpool L69 3GE, UK; 6 Institute of Clinical Neuroscience, University of Bristol and Southmead Hospital, North Bristol Trust, Bristol BS8 1QU, UK; kathreena.kurian@nbt.nhs.uk; 7 Department of Neurosurgery, Southmead Hospital, Bristol BS10 5NB, UK; Mario.Teo@nbt.nhs.uk; 8 Department of Neuropathology, Institute of Pathology, University Hospital Heidelberg, 69126 Heidelberg, Germany; Felix.Sahm@med.uni-heidelberg.de; 9 Clinical Cooperation Unit Neuropathology, German Consortium for Translational Cancer Research (DKTK), German Cancer Research Center (DKFZ), 69126 Heidelberg, Germany

**Keywords:** meningioma, atypical, benign, biomarker, plasma

## Abstract

There is an unmet need for the identification of biomarkers to aid in the diagnosis, clinical management, prognosis and follow-up of meningiomas. There is currently no consensus on the optimum management of WHO grade II meningiomas. In this study, we identified the calcium binding extracellular matrix glycoprotein, Fibulin-2, via mass-spectrometry-based proteomics, assessed its expression in grade I and II meningiomas and explored its potential as a grade II biomarker. A total of 87 grade I and 91 grade II different meningioma cells, tissue and plasma samples were used for the various experimental techniques employed to assess Fibulin-2 expression. The tumours were reviewed and classified according to the 2016 edition of the Classification of the Tumours of the central nervous system (CNS). Mass spectrometry proteomic analysis identified Fibulin-2 as a differentially expressed protein between grade I and II meningioma cell cultures. Fibulin-2 levels were further evaluated in meningioma cells using Western blotting and Real-time Quantitative Polymerase Chain Reaction (RT-qPCR); in meningioma tissues via immunohistochemistry and RT-qPCR; and in plasma via Enzyme-Linked Immunosorbent Assay (ELISA). Proteomic analyses (*p* < 0.05), Western blotting (*p* < 0.05) and RT-qPCR (*p* < 0.01) confirmed significantly higher Fibulin-2 (FBLN2) expression levels in grade II meningiomas compared to grade I. Fibulin-2 blood plasma levels were also significantly higher in grade II meningioma patients compared to grade I patients. This study suggests that elevated Fibulin-2 might be a novel grade II meningioma biomarker, when differentiating them from the grade I tumours. The trend of Fibulin-2 expression observed in plasma may serve as a useful non-invasive biomarker.

## 1. Introduction

Meningiomas are the most commonly occurring benign primary tumours of the central nervous system (CNS). They arise from the meningeal coverings of the brain and spinal cord, specifically from meningothelial (arachnoid cap) cells lining the arachnoid layer [1] or the Dural border cells found in the layer immediately superficial to the arachnoid layer [2].

According to the most recent (2016) World Health Organization classification of tumours of the central nervous system [3], there are three distinct histological tumour grades: the benign grade I meningiomas (70–85% of all meningiomas), the intermediate atypical grade II (15–30% of all meningiomas) and the most aggressive anaplastic/malignant grade III tumours (1–2%) [4,5,6]. Compared to the benign grade I tumours, the higher grade atypical and malignant meningiomas have a poorer prognosis with respect to mortality and recurrence free survival [7,8].

When diagnosing grade II tumours, inter-observer discordance can be as high as 12.2% (compared to 7 and 6.4% in grade I and III meningiomas, respectively) [9]. Their biological behaviour and risk of recurrence can range from indolent to highly aggressive despite being phenotypically identical [9,10,11,12,13], and their optimal management has not been defined and remains controversial [14,15,16,17,18,19,20,21].

Fibulin-2 is a large calcium binding extracellular matrix (ECM) glycoprotein hypothesized to stabilize and maintain ECM integrity and tissue architecture [22,23]. It interacts with other ECM proteins [24] known to be widely expressed in meningiomas such as MUC4 [25], α5β1 integrins [26], laminin-α2 chain and fibronectin, to facilitate cell motility, proliferation and angiogenesis [27], but is not currently known to play any role in meningioma pathogenesis. However, the Fibulin-2 protein has been described as a driver for malignant progression in lung adenocarcinomas [22,28], whereas the Fibulin 2 gene (FBLN2; chromosome 3p24-p25) has been reported to have tumour suppressive properties in nasopharyngeal carcinomas [23]. High FBLN2 expression is also known to be prognostic in liver (favourable) and in endometrial cancers (unfavourable) [28] (Human Protein Atlas available from http://www.proteinatlas.org, accessed on the 1st December 2020), and dysregulation of Fibulin-2 is involved in the development of breast cancer [24], Kaposi’s sarcoma and pancreatic cancer [22].

To the best of our knowledge, no previous study has investigated the clinical significance of Fibulin-2 as a meningioma biomarker. In this study, we identified Fibulin-2 as a novel biomarker for differentiating between grade II and I meningiomas and demonstrated higher expression in grade II meningioma (primary cells and tissue), at protein and gene expression levels. We also confirmed higher plasma Fibulin-2 concentrations in blood samples from grade II meningioma patients, compared to those from grade I meningioma patients.

## 2. Results

### 2.1. Fibulin-2 Is Significantly Differentially Expressed between Grade II and Grade I Primary Meningioma Cells

An extensive unbiased global protein expression (proteomic) analysis between grade I and II meningiomas was carried out via Mass Spectrometry (MS), using primary meningioma cell cultures derived from four grade I and four grade II tumours.

**Figure 1:**
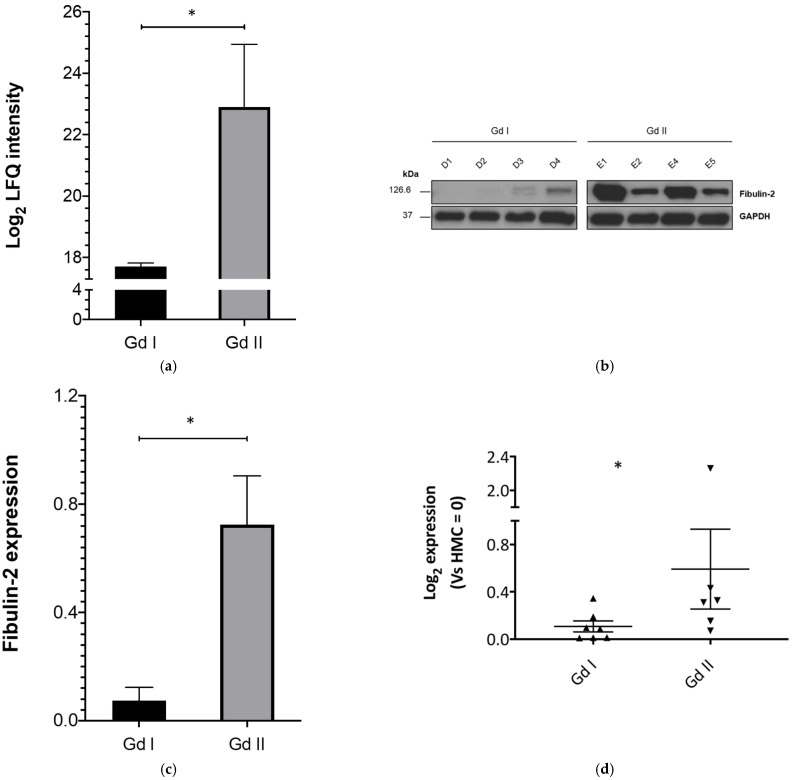
Fibulin-2 and FBLN2 are overexpressed/upregulated in grade II compared to grade I meningioma, in vitro. (**a**) Shows the mass spectrometry-derived Fibulin-2 comparative expression profile between grade I and II meningiomas; (**b**) representative Western blots of Fibulin-2 expression in grade I and II meningiomas; (**c**) shows Fibulin-2 expression in grade I (*n* = 4) and grade II (*n* = 4) meningiomas from the Western blot quantifications, normalized to GAPDH; (**d**) gene expression analysis by RT-qPCR of FBLN2 in meningioma primary cells. Data shown as Log 2-fold change (Fc) for grade I (*n* = 7) and grade II (*n* = 6) meningiomas. Data presented as mean ± SEM, * *p* < 0.05.

Of the 391 proteins identified (via MS) to be significantly differentially expressed between grades, Fibulin-2 expression was the most significant. It was observed to be overexpressed in grade II compared to grade I primary meningioma cells (Log_2_ Fc Gd II vs. Gd I = 5.19 and *p* = 0.02) (Figure 1a).

Using Western Blotting, we validated these findings on the primary cell cultures (discovery set) used during mass spectrometry analysis. This confirmed Fibulin-2 overexpression in grade II compared to grade I meningioma primary cells (Figure 1b,c).

Real-time Quantitative Polymerase Chain Reaction (RT-qPCR) gene expression studies on a validation sample set showed that FBLN2 gene expression was significantly higher (Log_2_Fc of Gd II vs. Gd I = 5.36; *p* < 0.05) in grade II (*n* = 6) versus grade I (*n* = 7) meningioma cells (Figure 1d).

### 2.2. Fibulin-2 Is Significantly Overexpressed in Grade II Compared to Grade I Meningioma Tissues

We further validated the findings from the primary cells experiments on a validation set of grade I and II meningioma tissues.

**Figure 2:**
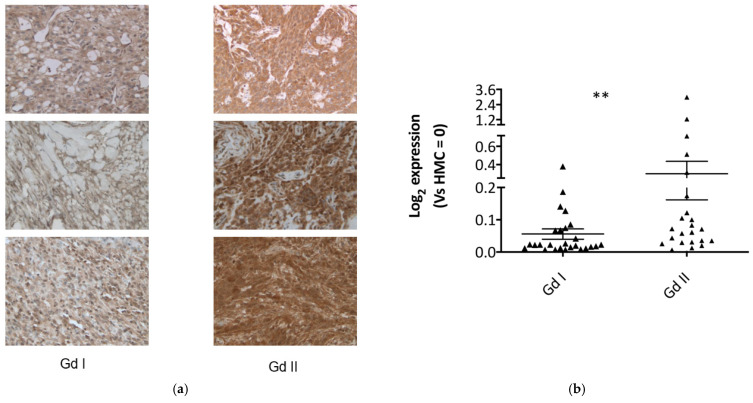
Fibulin-2 and FBLN2 are significantly overexpressed/upregulated in grade II compared to grade I meningioma tissue. (**a**) Representative immunohistochemistry images for Fibulin-2 staining in grade I and II meningiomas at 200× magnification. Staining was diffusely cytoplasmic in tumour cells, and more intense in the grade II meningioma sections; (**b**) gene expression analysis by RT-qPCR of FBLN2 in meningioma tissue is reported. Data shown as log 2-fold change (FC) for grade I (*n* = 26) and grade II (*n* = 23) meningiomas. Data presented as mean ± SEM, ** *p* < 0.01.

As an additional (semiquantitative) validation method, we carried out immunohistochemical studies on 10 different grade I tumour slides and 11 different grade II tumour slides, to compare expression between grades. Fibulin-2 staining was observed to be diffusely cytoplasmic in tumour cells, and more intense in the grade II tumours (Figure 2a), with 64% of grade II meningiomas staining strongly compared to 40% in the grade I cohort (Supplementary data S1).

In order to further evaluate the expression profiles of Fibulin-2 (FBLN2) in these meningioma grades, we carried out gene expression studies on 26 grade I and 23 grade II meningioma tumour tissues via RT-qPCR. This also revealed a higher FBLN2 expression (Log_2_Fc of Gd II vs. Gd I = 5.46; *p* < 0.01) in grade II meningiomas compared to grade I (Figure 2b).

These findings suggest that FBLN2 gene expression and Fibulin-2 protein levels are both increased in grade II meningiomas compared to the grade I tumours.

### 2.3. Plasma Fibulin-2 Levels Are Higher in Grade II Compared to Grade I Meningioma Patients

**Figure 3:**
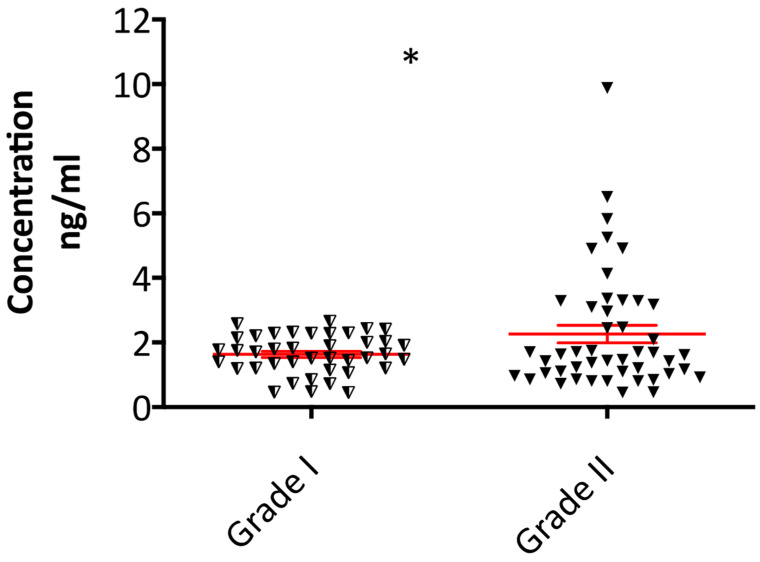
Higher plasma concentration of Fibulin-2 in grade II (*n* = 47) compared to grade I (*n* = 40) meningioma patients. Data presented as mean ± SEM, * *p* < 0.05.

The plasma levels of Fibulin-2 in patients with grade I (*n* = 40) and grade II meningiomas (*n* = 47) were assessed using ELISA, and shown in Figure 3. Compared to the grade I meningioma patients, Fibulin-2 plasma concentrations were significantly higher in grade II patients (*p* = 0.03).

When differentiating between grade II and I meningiomas, a Fibulin-2 blood plasma cut off value > 2.5 ng/mL has a 95% specificity for identifying patients with grade II meningiomas over those with grade I tumours.

**Figure 4:**
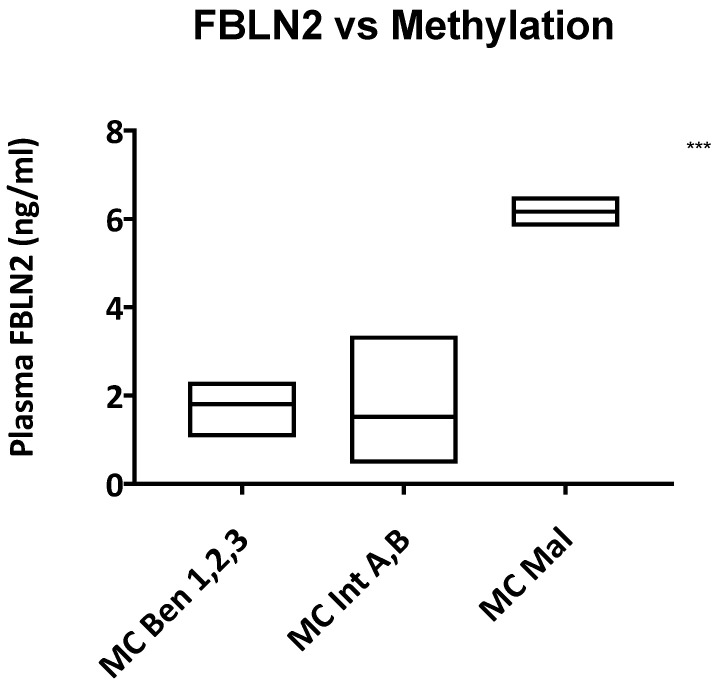
Plasma Fibulin-2 levels correlate with methylation class aggressiveness. The figure shows the relationship between methylation class and Fibulin-2 levels, irrespective of meningioma grade. MC = methylation class; Ben 1,2,3 = benign classes, Int A,B = intermediate classes, Mal = malignant class. ANOVA *** *p* < 0.0001.

We also found a significant correlation between high plasma fibulin-2 levels and more aggressive methylation classes, irrespective of meningioma grade (Figure 4).

## 3. Discussion

Meningiomas are among the most common primary tumours affecting the CNS and constitute between 30 and 35% of all primary CNS tumours [29,30], of which 15–30% are grade II tumours [4,6].

In this study, we assessed Fibulin-2 as a biomarker for differentiating between grade II and grade I meningiomas (Table 1), by evaluating its expression in meningioma cells, tissue and blood plasma levels.

A total of 178 grade I and II different meningioma cells, tissue and plasma samples (sometimes from the same patient) were used for the various experimental techniques employed to assess Fibulin-2 expression.

By performing MS proteomics validated with Western Blotting, we found that primary cells derived from grade II meningiomas expressed significantly higher levels of Fibulin-2 when compared to levels in grade I meningioma cells. RT-qPCR confirmed higher FBLN2 expression in the grade II meningioma cells.

We then assessed Fibulin-2 expression patterns in grade I and II meningioma tissue. IHC revealed more intense staining in the grade II tumours compared to grade I, while RT-qPCR showed that the FBLN2 gene is also overexpressed in grade II meningiomas compared to grade I, confirming the expression patterns observed in the meningioma cells. These findings are supported by our earlier proteomic study on meningioma tissue of different grades, where Fibulin-2 was identified to be non-significantly overexpressed in grade II meningioma tissue compared to grade I [12].

To assess the efficacy of Fibulin-2 as a non-invasive biomarker difference between grade I and II meningiomas, we carried out ELISA experiments on blood (plasma) samples from meningioma patients. We found significantly higher plasma concentrations of Fibulin-2 in grade II meningioma patients compared to the grade I cohort. However, there was a notable overlap in the spread of Fibulin-2 levels between grades, though we found that a cut-off value > 2.5 ng/mL was highly specific for patients with grade II meningiomas.

The observed overlap in grade I and II plasma Fibulin-2 levels was not surprising. Previous studies have described intra-tumoural biological heterogeneity in meningiomas [9,31,32] and a spectrum of biological behaviour in the grade II tumours [9,10,11,13], with biomarker validation studies often showing variable protein expression in these tumours, and similar expression profiles when compared to grade I and III meningiomas [12,33].

There are currently no reliable grade II-specific blood biomarkers, hence the objective of this study. We primarily focused on grade II tumours compared to grade I because the management of the grade I and III tumours is not as controversial as that of the grade II tumours, underlined by the ongoing debate surrounding the use of adjuvant radiotherapy in the management of grade II meningiomas [6,16,34,35].

Fibulin-2 has been shown to be involved in the physiological regulation of the development of the central/peripheral nervous systems [36,37], and its expression was observed to be increased at sites of traumatic CNS injury [38]. However, the mechanism underlying the Fibulin-2 expression patterns observed in this study remains unclear but may be related to the brain invasive properties of the higher grade meningiomas.

Fibulin-2 dysregulation is known to promote metastatic progression; mice studies have shown that Fibulin-2 is required for tissue repair following hypoxic stress [39] and is preferentially expressed in highly metastatic cells [40], while human transcriptomic studies have confirmed FBLN2 overexpression in metastatic tumours compared to the primary tumour site [41]. The study by Baird et al. on lung adenocarcinomas showed that high Fibulin-2 expression stabilizes the tumoural extracellular matrix (ECM) by acting as a biomechanical intermolecular anchor, thus driving malignant progression [23].

In this study, the observed increase in Fibulin-2 (FBLN2) expression in grade II meningiomas may be due to the protein stabilizing the tumoural ECM, promoting an environment that favours hypercellularity, cell proliferation [22] and (brain) invasion [42].

While this is primarily a diagnostic biomarker study, the mechanism underlying Fibulin-2 (FBLN2) expression in meningiomas will need to be investigated with further in vitro studies such as the knock-down of the FBLN2 gene and downregulation of Fibulin-2 expression in grade II meningiomas. The expression of the Fibulin-2 interacting proteins known to be expressed in meningiomas (MUC4, α5β1 integrins, laminin-α2 chain, type IV collagen and fibronectin) [22,24,25,26,27,43] can also be manipulated to assess the effects on the tumour biology (motility, invasion and proliferation) of grade II meningiomas, in order to better understand their heterogeneous histo-phenotypical characteristics, and discover more sophisticated biomarkers for clinical diagnosis/prognostication and potential molecular targets for treatment.

Review of our clinical data revealed a correlation between high plasma Fibulin-2 levels and a more aggressive meningioma methylation class, (Figure 4), but did not show a correlation between Fibulin-2 levels and patient demographics, radiological findings, histological characteristics or clinical outcome, meaning that the neuro-oncological use of Fibulin-2 should be in conjunction with these other factors to predict clinical outcome.

There are some limitations to this study. Expanding the study to include a larger sample cohort and longer (5–10 years) follow up period may improve the diagnostic accuracy of Fibulin-2 for differentiating between grade II and grade I meningiomas. A prospective study assessing preoperative and postoperative plasma Fibulin-2 levels will provide more data on its potential role as a biomarker for monitoring progression (in 19.5–46% of patients treated for a recurrence) [13,44] of grade I meningiomas or recurrence (in 39–58%) [6] of grade II meningiomas. We also suggest the evaluation of Fibulin-2 in grade III meningioma patients, though a large multicentre study will be required due to the small incidence/prevalence of grade III meningiomas.

In conclusion, we show that Fibulin-2 is expressed at higher levels both at the protein and RNA level, in grade II meningiomas compared to grade I. This study demonstrates that elevated Fibulin-2 levels might be a novel grade II meningioma biomarker, when differentiating them from the grade I tumours. The trend of Fibulin-2 expression observed in plasma from these patients may serve as a useful non-invasive biomarker when differentiating between both meningioma grades.

## 4. Materials and Methods

### 4.1. Clinical Material and Ethical Approval

The anonymized meningioma, Formalin Fixed and Paraffin Embedded (FFPE) and blood samples were obtained from the “UK Brain Archive Information Network (BRAIN UK)” biobank, under the ethical approval granted by the South West research ethics committee (REC No: 14/SC/0098; IRAS project ID: 143874, BRAIN UK Ref: 15/011); from the “Identification and validating molecular targets in low grade brain tumours (MOT)” biobank, under the ethical approval granted by the South West research ethics committee (REC No: 14/SW/0119; IRAS project ID: 153351; Plymouth Hospitals NHS Trust: R&D No: 14/P/056 and North Bristol NHS Trust: R&D No: 3458); and from the Walton Research Tissue bank (REC No: 15/WA/0385; IRAS project ID: 186041, WRTB Ref: 19_04).

All tumours were classified according to the 2016 WHO Classification of Tumours of the Central Nervous System.

Commercially available Human Meningioma Cells (HMC) cell lines were obtained from ScienCell™ and used as controls for the experiments involving primary meningioma cells or cell lines.

The clinical and demographic data for all the samples used are detailed in Supplementary data S2.

### 4.2. Meningioma Specimens, Tumour Digestion and Cell Culture

Meningioma specimens were collected during planned tumour resections; these are surplus samples destined for the incinerator following histological analysis for diagnosis and processed using techniques previously described [33,45].

Briefly, tumour (tissue) viability was maintained in a sterile transport media (DMEM supplemented with 10% FBS, 500 U/mL penicillin and streptomycin, 2.5 μg/mL amphotericin B) until processing. The tumour was then washed twice with sterile phosphate buffered saline (PBS, Gibco, Life Technologies, Loughborough, UK), transferred into incubation media (Dulbecco’s Modified Eagle Medium, DMEM (Gibco, Life Technologies, Loughborough, UK), supplemented with 10% FBS (Sigma Aldrich, Gillingham, UK) and 100 U/mL Penicillin/Streptomycin (Gibco, Life Technologies, Loughborough, UK)), and incubated at 37 °C in a humidified atmosphere (5% CO_2_) until digestion.

The tumours were then minced into small pieces and disaggregated in digestion media (Dulbecco’s Modified Eagle Medium, DMEM supplemented with 10% FBS, 100 U/mL Penicillin, 100 U/mL Streptomycin and 20 units/mL of Type III collagenase (Worthington Biochemical Corp, Lakewood Township, NJ, USA)) overnight at 37 °C. Following digestion, the cells were pelleted at 1500 rpm for 5 min, the supernatant was removed and the pellet re-suspended in the complete medium and seeded in the appropriate tissue culture dish [45].

The grade I primary cells used for Western blotting were cultured in DMEM supplemented with 10% FBS (Sigma Aldrich, Gillingham, UK), 1% D-(+)-glucose (Sigma Aldrich, Gillingham, UK), 100 U/mL penicillin/streptomycin (Gibco, Life Technologies, Loughborough, UK) and 2 mM GlutaMAXTM-I (Gibco, Life Technologies, Loughborough, UK) [33].

All the primary meningioma cells used for mass spectrometry and the grade II primary cells used for Western blotting were cultured in in Dulbecco’s Modified Eagle Medium F-12 Nutrient Mixture (Ham) (DMEM/F-12 (1:1)(1X) + GlutaMAX™-I; Gibco, Life Technologies, Loughborough, UK) supplemented with 20% FBS (Sigma Aldrich, Gillingham, UK), 1% D-(+)-glucose (Sigma Aldrich, Gillingham, UK) and 100 U/mL penicillin/streptomycin (Gibco, Life Technologies, Loughborough, UK).

The HMC cells were cultured in the recommended manufacturers’ Meningeal Cell Medium (Meningeal Cell Medium (MCM, Cat. #1401)).

All cell cultures were at 37 °C in humidified 5% CO_2_, keeping confluency above ~80%.

### 4.3. Western Blotting, Mass Spectrometry Proteomics and Immunohistochemistry

Protein extraction, quantification and cell culture Western blots were performed as previously described [12,33,45,46,47,48]. Antibodies used are listed in Supplementary data S3.

The proteomic analysis (on four grade I and four grade II meningioma primary cell cultures) was conducted according to previously described methods [12,46,47]. An amount of 50 μg of protein (cell lysates) was separated using SDS-PAGE on 4–15% Mini-PROTEAN^®^ TGX™ Precast Protein Gels (Bio-Rad). The gels were stained with colloidal Coomassie blue stain (Life Technologies) until lanes were visible, then de-stained with de-staining solution.

Sample lanes were cut out and sliced into 10 fractions that were further diced into 1 mm^3^ pieces before in-gel digestion according to the Shevchenko protocol. The samples were cleaned and desalted by STAGE tips (made in-house) as previously described [47] and re-suspended in 0.5% acetic acid, 1% trifluoroacetic acid (TFA) in a final volume of ~25 µL for mass spectrometry analysis.

Mass Spectrometry and protein identification were performed according to protocols previously described [12,46,47].

For immunohistochemical (IHC) studies, 4 μm FFPE sections from 21 (10 grade I and 11 grade II) tumours were processed using the method previously described by our lab [12,33,48]. Following EDTA antigen retrieval, the sections were further blocked using normal horse serum and the Avidin-biotin blocking solution to reduce non-specific signal [48], prior to incubation with the primary antibody (Supplementary data S3).

The slides were visualised with the Vectastain Elite ABC-HRP kit (Vector Laboratories Ltd, Burlingame, USA) according to the manufacturer’s protocol. For control, sections were incubated without addition of the primary antibody [12,33,48].

The immunohistochemical results were reviewed “blind” to histological grade by a neuropathologist (D.H). Semiquantitative assessment of the intensity of immunoreactivity was undertaken and scored as follows: 0 none; 1 weak; 2 moderate; 3 strong (Supplementary data S1).

### 4.4. RNA Isolation and Gene Expression Analysis

Total RNA was extracted from 62 frozen tissues and cells using the Qiazol reagent (Qiagen UK), according to the manufacturer’s protocol. The ThermoFisher Scientific Nanodrop 2000 Spectrophotometer (Waltham, MA, USA) was used to assess the quality and concentration of the RNA [33,45].

Real time polymerase chain reaction (RT-PCR) was performed on 1 μg of total RNA using the High-Capacity cDNA Reverse Transcription Kit, according to the manufacturer’s instructions.

qPCR was performed on a LightCycler 480 Real-Time system (Roche), with TaqMan^®^ probes (FBLN2 ID Hs00157482_m1 and Glyceraldehyde 3-phosphate dehydrogenase, GAPDH ID Hs02786624_g1 [Applied Biosystems, ThermoFisher Scientific, Loughborough, UK]) according to the manufacturer’s protocol, in triplicate for each gene.

GAPDH was used as internal control, RNA extracted from the HMC cell line was used as a calibrator and the 2^−(ΔΔCt)^ method was used for relative gene expression quantification [33,45,49].

### 4.5. Enzyme-Linked Immunosorbent Assay

A commercially available (abx350725, Abbexa Ltd., Cambridge, UK) sandwich enzyme-linked immuno-sorbent assay (ELISA) for the quantitative measurement of plasma Fibuin-2 levels was used according to the protocol (https://www.abbexa.com/human-fibulin-2-elisa-kit-2, accessed on the 1st December 2020) provided by the manufacturer, to compare levels in grade I and II meningioma patients.

The plasma samples were derived from blood collected (pre-operatively) in lavender BD Vacutainer^®^ EDTA tubes (Becton Dickinson U.K. Ltd., Swindon, UK). The samples were gently mixed (by inversion) for a minute, then centrifuged at 2400× *g* for 10 min at 4 °C. The supernatant was collected and stored at −80 °C pending the ELISA experiments.

### 4.6. Methylation Profiling

DNA was extracted from tumour tissue using the Qiagen DNeasy Blood and Tissue DNA extraction kit (QIAGEN, Manchester, UK-Cat No: 69504), with the (extracted) DNA concentrations calculated using the ThermoFisher Scientific Nanodrop 2000 Spectrophotometer [45]. Methylation profiling was performed using the Infinium MethylationEPIC (850k) BeadChip (Illumina, San Diego, CA, USA) or Infinium HumanMethylation450 (450k) BeadChip array (Illumina) as previously described [50].

### 4.7. Statistical Analysis

According to the experimental procedure, probability (*p*) values were estimated with the Student’s *t*-Test or the ANOVA one-way analysis of variance, using the GraphPad Prism 8.4.2 and Microsoft Excel 2016 software programs. *p* values < 0.05 were considered to be statistically significant. The results are expressed as the mean ± standard error of the mean (SEM).

## Figures and Tables

**Table 1:** Clinical characteristics of the meningioma samples used (*n* = 178). There were two grade I meningioma patients with mixed histology; one had metaplastic, transitional and microcystic (sphenoid wing) and transitional (frontal) tumours; the other had a mixed meningothelial, microcystic and angiomatous meningioma.

Clinical Features	Group	Patients	
		*n*	(%)
Sex	Female	123	69.1
	Male	54	30.3
	Unknown	1	0.6
Age	Median Range	59 27–92	
WHO grade	WHO I	87	48.9
	WHO II	91	51.1
Grade I	Meningothelial	27	31.0
	Fibrous	25	28.7
	Transitional	17	19.5
	Psammomatous	6	6.9
	Angiomatous	1	1.1
	Secretory	1	1.1
	Metaplastic	1	1.1
	Not reported	6	6.9
	Meningothelial + bony invasion	1	1.1
	Mixed	2	2.3
	Chordoid	7	7.7
	Atypical	71	78.0
	Clear cell	1	1.1
	Atypical + brain invasion	11	12.1
	Atypical + bone/dura invasion	2	2.2
	Chordoid + dura invasion	1	1.1
Grade II	Atypical with extracranial extension	1	1.1
	Atypical + focal rhabdoid features	1	1.1
Location	Falcine	10	5.4
	Cerebello-pontine angle	4	2.2
	Parasagittal	15	8.1
	Olfactory groove	11	5.9
	Frontal	42	22.7
	Occipital	17	9.2
	Intraventricular	2	1.1
	Spheno-orbital	3	1.6
	Sphenoid wing	9	4.9
	Tentorial	4	2.2
	Parietal	9	4.9
	Temporal	9	4.9
	Posterior fossa (not specified)	9	4.9
	Planum sphenoidale	6	3.2
	Fronto-parietal	13	7.0
	Fronto-temporal	2	1.1
	Petrous temporal (incl. petro-clival)	3	1.6
	Cavernous sinus	1	0.5
	Planum sphenoidale	6	3.2
	Thoracic	2	1.1
	Skull base (not specified)	1	0.5
	Not specified/stated	7	3.8

## Supplementary Materials

Supplementary Materials can be found at https://www.mdpi.com/1422-0067/22/2/560/s1. Supplementary data S1—Immunohistochemistry staining intensity scores; Supplementary data S2—detailed clinico-demographic and plasma Fibulin-2 concentration data; Supplementary data S3—Antibody details.

## Abbreviations

CNS	Central Nervous System
CDKN2A/B	Cyclin-Dependent Kinase Inhibitor 2A/2B
ECM	Extra-cellular Matrix
EDTA	Ethylenediaminetetraacetic acid
ELISA	Enzyme Linked Immuno-Sorbent Assay
FBLN2	Fibulin-2 gene
Fc	Fold change
FFPE	Formalin-Fixed, Paraffin-Embedded
GAPDH	Glyceraldehyde 3-phosphate Dehydrogenase
Gd I	Grade I meningiomas
Gd II	Grade II meningiomas
MS	Mass spectrometry
MUC4	Mucin 4
NF2	Neurofibromatosis Type 2
SDS-PAGE	Sodium Dodecyl Sulfate–Polyacrylamide Gel Electrophoresis
RT-PCR	Reverse transcriptase polymerase chain reaction
RT-qPCR	Reverse transcriptase quantitative Polymerase Chain Reaction
WHO	World Health Organization
