# Supplementary material for: Fibulin-2: A Novel Biomarker for Differentiating Grade II from Grade I Meningiomas
Source: Int J Mol Sci. 2021 Jan 8;22(2):560. doi: 10.3390/ijms22020560 (PMC7827565; doi:10.3390/ijms22020560)
Supplement: Supplementary file 1 [file ijms-22-00560-s001.zip › Supplementary Files/Supplementary data S3.docx]

**Supplementary data S2**. Details of the antibodies used.

A. Western blotting antibodies

| **Primary antibody** | **Species** | **Company** | **Catalogue No.** | **Dilution** |
| --- | --- | --- | --- | --- |
| Fibulin-2 | Rabbit | ThermoFisher Scientific | #PA5-21640 | 1:500 |
| GAPDH | Mouse | Millipore | MAB374 | 1:50,000 – 1:100000 |

| **Secondary antibody** | **Type** | **Company** | **Catalogue No.** | **Dilution** |
| --- | --- | --- | --- | --- |
| Goat anti-Rabbit | HRP-conjugated | Bio-Rad | #170-6516 | 1:3000 |
| Goat anti-Mouse | HRP-conjugated | Bio-Rad | #172-1019 | 1:3000 |

B. Immunohistochemistry

| **Antibodies** | **Pretreatment** | **Company** | **Catalogue No.** | **Dilution** |
| --- | --- | --- | --- | --- |
| Fibulin-2 | 30 min EDTA | ThermoFisher Scientific | #PA5-21640 | 1:200 |
